# Using linked administrative data to evaluate and improve the quality of end-of-life care in nursing homes.

**DOI:** 10.23889/ijpds.v7i3.2007

**Published:** 2022-08-25

**Authors:** Elizabeth Pereira

**Affiliations:** 1 Office for National Statistics

## Objectives

To analyse the linked dataset between England and Wales 2021 Census/Census Coverage Survey and the Demographic Index (DI), would provide insights on quality and coverage, enabling improvements of using admin data to form population/ household estimates for future linkages and to evidence additional admin sources needed to capture the population.

## Approach

The DI is a composite dataset made up of several linked admin sources. Therefore, the linked output is complex, made up of clusters where individual sources within the clusters have been linked to the Census using the different available information across sources. To carry out the analysis, a flagging strategy has been designed to enable analysts to form cuts of the linked dataset that are specific to their research needs. High level research questions have been designed to provide fast paced analysis to inform the National Statistician’s 2023 recommendation on the need for a future Census.

## Results

Providing insight into how to improve the current linkage methodology of the DI
How we use the DI to construct Statistical Population Datasets (which use inclusion rules to establish the usual resident population)
Identifying the extent of over and under coverage in the Statistical Population Datasets, allowing development of an appropriate estimation strategy to more accurately estimate the population

The results will also inform how we utilise linked composite data sources in the future, as we can share the lessons learnt in how we planned to use the data, including our flagging strategy verses the reality faced when undertaking the analysis.

## Conclusion

This analysis will inform approaches to using linked composite data in the future but also provide a wealth of knowledge including: informing linkage methods, defining the population estimation challenge and providing insights into how we deliver admin based population estimates.

